# Protective Effect of Aortic Stenosis on the Coronary Arteries. Hypothetic Considerations to an Old Enigma

**DOI:** 10.5935/abc.20160039

**Published:** 2016-04

**Authors:** Paulo Roberto Barbosa Evora, Livia Arcêncio, Alfredo José Rodrigues, André Schmidt

**Affiliations:** Faculdade de Medicina de Ribeirão Preto - Universidade de São Paulo, São Paulo, SP - Brazil

**Keywords:** Aortic Valve Stenosis, Coronary Artery Disease, Coronary Angiography

## Abstract

A literature overview of angiographic studies has shown that the prevalence of significant coronary disease in patients with aortic stenosis (AS) varies from 20 to 60%. Early necropsy studies suggested that patients with AS had a lower than expected incidence of coronary artery disease (CAD), originating the concept of a protective effect of AS on the coronary arteries. The myth of AS protection against CAD would be better explained as endothelium-myocardial interaction (crosstalk) protection triggered by left ventricular overload. Therefore, the cGMP/NO pathway induced by the AS overload pressure would explain the low incidence of CAD, which is compatible with the amazing natural long-term evolution of this cardiac valve disease.

## Introduction

An overview of literature angiographic studies has shown that the prevalence of the significant coronary disease in patients with aortic stenosis (AS) varies from 20 to 60%. Early necropsy studies suggested that patients with AS had a lower than expected incidence of coronary artery disease (CAD), originating the concept of a protective effect of AS on the coronary arteries.^1,2^

Some publications illustrate this concept. Among 88 patients with AS requiring valve replacement at Hammersmith Hospital, twenty-two (34%) had significant CAD (diameter < 50%).^3^ Morrison et al.^4^ analyzed coronary arteriograms of 239 patients investigated for valvular heart disease during a five-year period. Significant CAD was present in 85% of patients with mitral valve disease and in only 33% of patients with aortic valve disease. There was, however, a significant inverse association between CAD severity and valve disease severity in patients with aortic valve disease.^4^ A total of 574 patients with severe AS (mean age of 65.9 ± 9.6 years) were assessed in a Korean study, with significant CAD being reported in 61 patients (10.6%). There was a low incidence of significant CAD in a population of Korean patients with severe AS. Coronary angiography before AVR was considered in patients with multiple cardiovascular risk factors, or in patients older than 69 years without risk factors.^5^

A retrospective observational Mayo Clinic study suggests that coronary artery bypass grafting (CABG) associated with AVR has similar operative mortality, albeit with improved overall survival during the long-term follow-up in patients undergoing AVR without CABG.^6^ However, a large Society of Thoracic Surgeons database study demonstrated that the addition of CABG to AVR increased surgical morbidity and mortality, raising the critical conjecture that revascularization might have an impact on long-term survival. Also, the most recent American Heart Association and American College of Cardiology guidelines^7^ downplay the importance of CABG at the time of surgical AVR and the indication for revascularization in patients with coronary artery lesions > 70% has been downgraded from a class I to a class IIa indication, minimizing the importance of 50% to 70% stenotic lesions.^8^

Based on these literature data, some key points are clearly established:

1. Early necropsy studies suggest that patients with AS had a lower CAD incidence.^1,2^
2. Significant CAD was present in 85% of patients with mitral valve disease and angina, but in only 33% of patients with aortic valve disease and angina.^3-6^
3. A Society of Thoracic Surgeons database study demonstrated that the addition of CABG to AVR increased surgical morbidity and mortality.^7,8^
4. The most recent American Heart Association and American College of Cardiology guidelines downplay the importance of CABG at the time of surgical AVR and the indication for revascularization in patients with coronary artery lesions greater than 70% has been downgraded from a class I to a class IIa indication, deemphasizing the importance of 50% to 70% stenotic lesions.^7,8^
5. Transcatheter aortic valve implantation (TAVI) changed the guidelines for AS in patients with high comorbidity, without any consistent rule, concerning CABG in the presence of moderate CAD. While CABG may favorably influence the long-term outcome in patients undergoing surgical implantation of aortic prosthesis, this information is not yet applicable to TAVI, because it has not been possible to establish the profile of its long-term outcome.^6^ Many patients who have severe AS have angina without CAD, and both can be free of angina with valve replacement. This information is very important, considering the advent of Transcatheter Valves.

**Figure 1:**
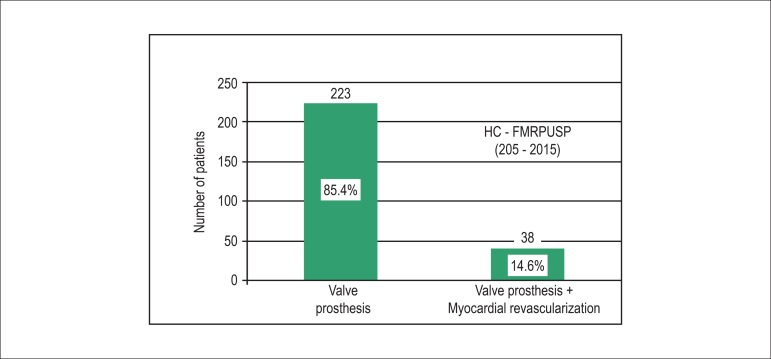
Aortic valve prosthesis associated or not with myocardial revascularization at Faculdade de Medicina de Ribeirão Preto, Universidade de São Paulo SP, Brazil (2005 - 2015) (isolated aortic valve stenosis, after excluding congenital aortic stenosis and bicuspid aortic valve).

The myth (Paradigm? Mistery? Puzzle?) of AS protection against CAD is still impossible to overlook. There is no hypothesis, or even speculation about the small incidence of severe CAD in association with AS. For the present text we performed an analysis of the national data, which confirmed the worldwide data (Figure 1).

The first relevant information was the well-demonstrated fact that in ventricular hypertrophy secondary to chronic systemic hypertension or aortic valve disease, coronary diameters are increased, as documented by Kimball et al.^9^ In 32 patients with AS, the coronary artery luminal diameters were compared with those of 24 control subjects without LV hypertrophy using a derived index. Patients with AS had significantly larger coronary arteries than the control subjects.^9,10^ In patients with AS, LV hypertrophy progression is associated with left anterior descending and left circumflex coronary artery increased dimensions, whereas the right coronary artery remains unchanged. It is interesting to mention that despite the enlargement of the left coronary artery, its cross-sectional area per 100 g of LV muscle mass decreased. Hence, the increase in coronary artery size appears to be inadequate when LV hypertrophy severity increases. Another interesting observation is that left coronary artery size decrease after valve replacement at an equal rate with LV muscle mass regression. Also, enlargement of the coronary arteries has been reported in patients with LV hypertrophy at necropsy and in clinical studies of patients with aortic valve disease who were not yet candidates for surgery. As time goes by, the severity of aortic valve stenosis is accompanied by significant hypertrophy, growing increase in left coronary artery dimensions, and no changes in the right coronary artery.^11^

At this point we have to add other key points, in an attempt to obtain some clues to establish some hypotheses:

1. Increased coronary diameters are systematically observed in association with ventricular hypertrophy secondary to chronic systemic hypertension or aortic valve disease.
2. In patients with aortic valve stenosis, LV hypertrophy progression is associated with an increase in left coronary dimensions, while right coronary artery dimensions remain unchanged.^9-11^
3. Coronary artery size increase seems to be insufficient when LV hypertrophy severity increases.^9-11^
4. An enlarged left coronary artery size in the preoperative period, decreases after valve replacement at an equal rate with the LV muscle mass regression.^11^
5. As time goes by, aortic valve stenosis severity increases in association with significant LV mass increase, a further increase in left coronary artery dimensions, whereas those of the right coronary artery remains unchanged.^11^

These data were concisely presented by Kauffman et al.^12^: 1) Coronary artery size increases as LV mass increases in both primary and secondary hypertrophy. 2) The enlargement of left coronary cross-sectional area is independent from the cause of LV mass increase. 3) Coronary artery dimensions are inappropriate concerning LV hypertrophy. Thus, the stimulus for coronary artery growth is not influenced by the underlying disease, but seems to depend on the LV hypertrophy degree.^12^

"These data allow for a pivotal conclusion: The association of coronary enlargement is clear, emphasizing the phenomenon that is present only in the left hypertrophic ventricle and resulting in pressure overload, as the coronary artery size remains decreased after the aortic valve prosthesis implant".

The next step was to direct our attention to the microvasculature, endothelium function, and nitric oxide. Changes in the microvasculature could lead to a decrease in coronary flow reserve and thus could be associated with the inadequate growth of the epicardial coronary arteries. However, it has been shown in patients with aortic valve disease that coronary flow reserve tends to normalize after successful valve replacement, suggesting that the microvasculature is not altered by hypertrophy and is not associated with an increase in the microvascular bed cross-sectional area.^11^ Therefore, using logical thinking, myocardial hypertrophy would be involved in the pressure overload.

Endothelial regulation of vascular activity by relaxing and contracting factors has been well established. Experimental evidence suggests a similar modulation of myocardial contractile performance by endocardial and coronary vascular endothelium.^13^ The human heart has a plurality of cell types, with fibroblasts and other connective tissue cells being the most abundant. The remaining cell mass consists of cardiomyocytes (CM), endothelial cells (EC), smooth muscle cells, mast cells, and immune-related cells. CM are surrounded by the dense capillary network, which is critical for maintaining constant blood flow.^14^ The several studies along this line of research allow us to consider the concept of EC-CM crosstalk. Several failed clinical studies targeting cell-cell interactions emphasize the need to understand the molecular interactions between various cells *in situ.*

**Figure 2:**
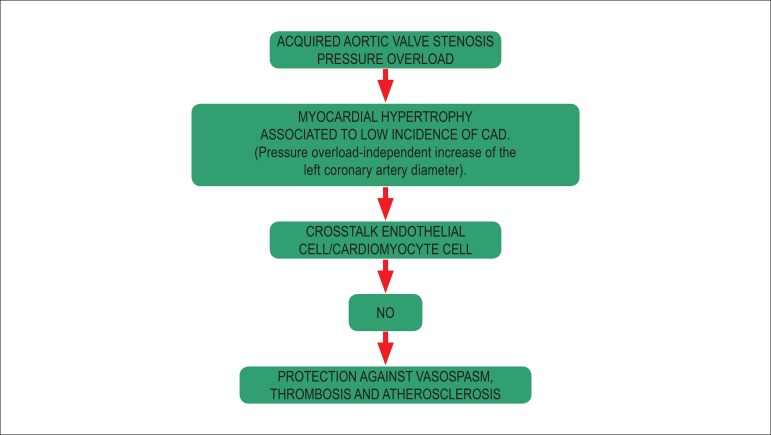
Physiopathological suggestion for the small incidence of coronary artery disease and natural history (> 50 years without symptoms) in patients with acquired aortic valve stenosis.

In conclusion, the myth of AS protection against CAD would be better presented as endothelium-myocardial interaction (crosstalk) protection triggered by left ventricular overload. Therefore, the cGMP/NO pathway induced by the AS overload pressure would explain the low incidence of CAD, which is compatible with the amazing natural long-term evolution of this cardiac valve disease (Figure 2).
